# A feasibility study of 60 consecutive patients operated for unstable thoracic cage

**DOI:** 10.1186/s13032-014-0020-z

**Published:** 2014-12-30

**Authors:** Hans P Granhed, David Pazooki

**Affiliations:** Department of Surgery, Sahlgrenska University Hospital, Gothenburg, Sweden

**Keywords:** Rib fractures, Flail chest, Treatment, Operation

## Abstract

**Background:**

About 10% of adult patients in high-energy trauma sustain multiple rib fractures. Some of these patients suffer from flail chest leading to respiratory insufficiency. During last years interest and results for operative treatment has improved. The literature today all show positive results for surgical versus conservative treatment, specifically with regard to time spent in mechanical ventilator, complication rates and length of hospital stay.

**Methods:**

Between September 2010 and July 2012, 60 patients with flail chest or multiple rib-fractures resulting in unstable thoracic cage were operated. 16 women and 44 men with an age between 19-86 years (mean 57). We used modern fracture techniques with plates and intramedullary splint. Thoracotomy was performed and lung lacerations were debrided (11/60). Time spent in ventilator, complications and other adverse effects was studied. The operated cohort was compared to results from six previous years, when none was operated for that diagnose (153 patients).

**Results:**

There is a significant correlation between Injury Severity Score (ISS) and time spent in ventilator both for patients operated and not operated (p< 0,01). The mean time in ventilator was 9,01 days for not operated patients compared to 2,7 for the operated (p<0,0001). No clear pneumonias were found. We had two deaths during the acute period. The infection rate was low.

**Conclusions:**

Open reduction and internal fixation is a safe method to treat the unstable thoracic cage with multiple rib fractures and flail chest. Complication are few. The treatment time in mechanical ventilator is significant decreased. The operative treatment is probably cost effective and can be recommended. Knowledge in thoracic surgery and modern fracture surgery is needed. This is a therapeutic consecutive, level III, cohort study with historical controls.

## Introduction

About 10% of adult patients in high-energy trauma suffer from multiple rib fractures [1]. According to our trauma register (KVITTRA) [2], the incidence among adults in our region was 9%. Paediatric multiple rib fractures are seldom caused by trauma but are frequently related to child abuse [3].

The prevalence of multiple rib fractures in geriatric patients rarely receives attention proportional to that given to patients of younger ages, despite mortality rates between 10 and 30% from complications [4].

Pavalen et al. recently concluded that, because of the increasing length of life expectancy, the prevalence of multiple rib fractures will increase during the next decades [5,6].

Bemelman et al. presented a historical review in 2010 of the different surgical treatments of multiple rib fractures and flail chest dating from the 1940s to the present [7].

A more current and extensive review article by Lafferty et al. was published in 2011 [8] where the focus was current clinical practice and inclusion or exclusion criteria of patients regarding surgical versus conservative treatment of multiple rib fractures.

To date, few randomized, single-centre, controlled studies have been conducted, but those that have been carried out show positive results for surgical versus conservative treatment, specifically with regard to time spent in a mechanical ventilator, complication rates, length of hospital stay and return to full time employment [9,10]. In a retrospective health economy study, Bhatnagar et al. found a positive effect of surgical treatment [11].

A Health Technology Assessment (HTA) was made before we started this study. We found that there is evidence in favour of surgical treatment of patients with flail chest and impaired ventilation compared to treatment with mechanical ventilation. The level of evidence is however low [12]. We also compared the direct cost during primary treatment and found that the costs for operated patients were slightly lower [12].

In 2011, Althausen et al. published a retrospective case–control study with similar implants and concluded that locked plate fixation appears to be favourable and had few complications [13]. Three-dimensional reconstructions from CT are an important assessment before surgery to visualize the extent of the damage to the thoracic cage and the subsequent deformity [14,15].

This is a consecutive feasibility study of traumatic thoracic cage instability treated with open reduction and internal fixation that can be compared with historical controls from the same hospital and area in previous years.

The aim of this study was to assess possible, benefits, adverse effects and early complications in operative treatment using the principles of modern fracture surgery techniques and specially designed titanium implants (Matrix®) in combination with advanced 3D reconstructions based on modern CT scanning. We compared the prospective results with historical controls.

## Patients and methods

Our hospital receives about 1000 high-energy emergency trauma patients per year. These patients have either impaired vital parameters (red alert) or have been involved in a high-energy trauma but still have normal vital parameters (orange alert). Trauma patients are prospectively recorded in the historical database, “KVITTRA”.

The study was approved by the Regional Ethical Review Board (Dnr 053-12). From January 2005 until December 2010 762 out of 8680 (9%) trauma patients had rib fractures. 153 (20%) out of these 762 also needed mechanical ventilator due to respiratory insufficiency. These 153 were used as a non operated reference group retrieved from the historical database.

**Figure 1:**
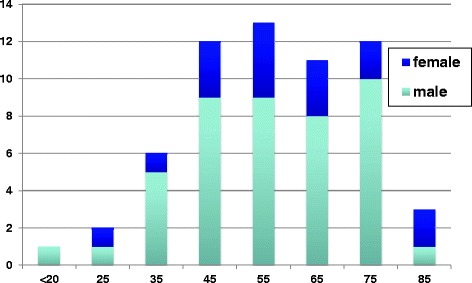
**Age and gender (n = 60).**

Between September 2010 and July 2012 60 patients were included in the prospective study (Figure 1). All patients in the prospective study had impaired saturation in spite of oxygen administration and were suffering from severe pain. Fifty-six patients had segmental fractures in three or more adjacent ribs, leading intermediate flail segments. The other four had nine fractured ribs or more resulting in impaired respiration as a direct mechanical effect or incurable pain.

A new implant based on locked screws in low profile, pre shaped titanium plates and IM splints was used (MatrixRIB Fixation System, (Sythes Inc. 2014)). This system applies the concept of angular locked plates and intramedullary nailing and has been thoroughly tested [16].

**Figure 2:**
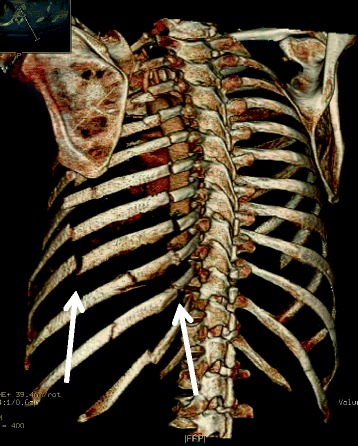
**Multiple flail segments after a car crash.**

All patients were intubated with a double luman tube. Prophylactic antibiotics were given to all patients until the drains were extracted on day three or four. The affected lung was emptied during at least part of the surgery depending on the patient’s ability to saturate on one lung. The surgery was performed in all cases by experienced trauma surgeons with a special interest in thoracic or fracture surgery. The surgical tactics were planned on the basis of 3D reconstructions (Figure 2). Most patients were operated in the side position with a lateral thoracotomy incision, but often curved around the scapula. The entry between the ribs was usually done between two of the most damaged ribs. Leakage of blood and air was fixed by sutures but if the lung tissue was lacerated, small non anatomic resection was also performed, with staples. The fractured ribs were then stabilized with the MatrixRIB Fixation System. If the intermediate segment was short, the double fractures were fixed with a continuous long plate with the bridging technique [17]. Standard plates, in compression, were used on single fractures or long intermediate segments [18]. “Stable fractures”, especially posterior, and fractures in the upper three ribs were left or fixed with the splints or “sewn” together with a modified Robiscek technique [19]. Matrix IM splints do not offer the same stability as plates and were therefore avoided for adlatus dislocated ribs. They were mainly used when the exploration was hindered by the scapula. All implants were positioned on the outside of the thorax.

One drainage was introduced in the front and one in the back side of the lung. In some cases, we also introduced an epidural catheter into the pleura to add local anaesthesia.

The thoracic cage was closed with three or four strong, double, resorbable sutures around stable, properly fixated ribs. The soft tissue was then closed in layers. After surgery, the patients were extubated according to the anaesthesiologist in the theatre or ICU. Chest x-rays in two plains were taken during the first week. The times in the hospital and ventilator, general complications and other adverse effects were monitored.

Criteria for extubation were: less than 40% oxygen, pressure at inspiration and expiration less than 8 cm water, respiratory frequency below 25 and patient wake and alert.

### Data collection and statistics

Data from the 60 first operated patients were continuously entered in a database.

Patients in our trauma base (KVITTRA) fulfilling the criteria of high-energy trauma, rib fractures and possible or prompt need for mechanical ventilation because of respiratory insufficiency were included in the study and used as a non operated reference group.

SPSS version 20 (IBM Corporation, Somers, NY) was used for statistical analyses. Pearson’s correlation was used between ISS and ventilated days. The t-test was used for differences in ISS and days spent in a mechanical ventilator.

Direct costs were registered for each patient. The costs for the patients retrieved from the historial database were corrected for index.

Only the first 29 patients could be analysed for costs. Thirty-nine similar patients from the control group were used as controls. These two groups were however not analysed for age, gender and ISS.

## Results

The dominant injury mechanism was a high-energy trauma falling from heights (33%), motorcycle accidents (24%) and car accidents (20%).

Only five patients had isolated thoracic injuries; all others had multiple injuries. The mean Injury Severity Score (ISS) was 21.7 (SD +−10.8) in 47 patients. Thirteen patients were referred from other hospitals, and the ISS was therefore not calculated in our systems. The other 145 injuries and the corresponding 62 surgeries are listed in Table 1. For amount of rib fracture surgery see Table 2.

Ten patients were referred to our clinic between 24 and 48 hours after injury. Three patients were referred to us because of unstable spine but also flail chest; in these cases, they were operated in the supine position before receiving spine surgery in the prone position. All other patients came from the emergency department and were taken care of according to the ATLS concept.

Fifty-six patients fulfilled the criteria of flail chest: three segmental fractures leading to a flail segment. The other four had multiple rib fractures resulting in impaired respiration as a direct mechanical effect or incurable pain.

**Table 1:** **Other injuries and surgery**

**Diagnose**	**Number**	**Surgery**
Pelvis fracture	11	6
Difragm rupture	7	7
Lung cuntusion	45	11
Liver injury	7	2
Spleen rupture	7	6
ruptured bowel	1	1
Uncontrolled bleeding	1	Emergency thoracotomi
Mangled extremity upper	1	amputation
Mangled extremity lower	1	amputation
Lumbar spine fx.	6	6
Thoracic spine fx.	4	4
Sternum fx.	5	0
Commotio cerebri	9	0
Fracture to the skull	6	4 icp.
Subdural bleedeing	5	3
Subarachnoidal bleeding	5	2
Face fracture	22	3
Upper exstremity fx.	1	10
Lower extremity fx.	1	1

**Table 2:** **Rib surgery**

	**Number**	**Spread**	**Mean**
fractured ribs	449	2-14	7,5
fixated ribs	336	2-10	5,6
plates	222	0-9	3,7
cerclage	109	0-5	1,8
im rods	33	0-3	0,2

Sixty patients with flail chest or multiple rib fractures resulting in an unstable thoracic cage were given surgery between September 2010 and July 2012. Respiratory insufficiency was the main cause of surgery in 46 of the patients. Twenty-two patients also needed a thoracotomy because of uncontrolled bleeding or air leakage, which added to the indication for open surgery. During this period, all patients that fulfilled the criteria mentioned above were operated and followed until discharged from hospital.

**Figure 3:**
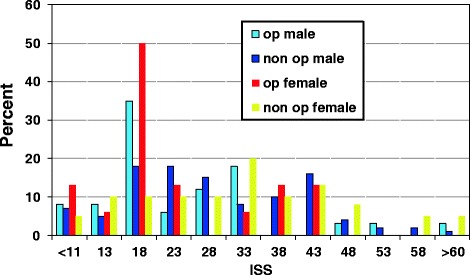
**Frequency of patients from the two series and gender compared to ISS.**

There were 16 women and 44 men aged between 19 and 86 years (mean 57 years). Three patients had significant COPD and three patients had emphysema at the time of injury. The distributions of age and gender are shown in Figure 1 and age and gender in percent compared to ISS in Figure 3.

The median day for operation was day four (1–59) after trauma. The left thorax was operated in 26 cases and the right in 34 cases. Bilateral fixation was performed in one case. The number of ribs and fixations is reported in Table 2.

The thoracotomy was performed in all cases to clean out the pleura. Leaking lung parenchyma was sutured in seven cases and lacerated lung parenchyma was also resected in ten cases as a non anatomic or tract resection with staples. One case had a totally occluded and non-functioning inferior lobe that was resected. Pleurodesis was performed in 11 cases. Seven cases of ruptured diaphragm were found and in all cases sutured.

The number of fractured ribs varied from three to 14, with a mean of 6.3.

Significant haemothorax was present in 61% and pneumothorax in 63%. All of these patients had at least one chest drain as an emergency procedure. Seventy-five per cent had pulmonary contusion at primary CT.

### Historical controls

From January 2005 until December 2010 762 out of 8680 (9%) trauma patients had rib fractures. 153 (20%) also fulfilled also the other criteria above. The mean ISS for these 153 patients was 30.9 (SD+−13.3). The mean time in the mechanical ventilator was 9.0 days and the median stay was 5 (range 1–176) days. There was a significant correlation between ISS and days in ventilator median (p<0.007).

### Time in mechanical ventilation and ISS

**Figure 4:**
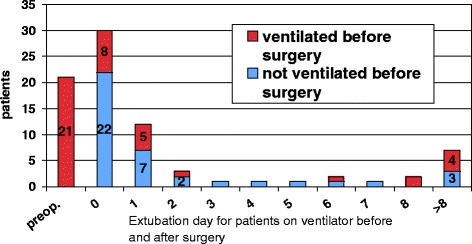
**Day of extubation for patients on ventilator before surgery and those who did not yet need mechanical ventilation before surgery.**

The mean time in the ventilator in operated patients was 2.7 days and the median 0.5 (range 0–21) days, and the ISS was 21.7 (SD+−10.7). Before surgery, 21 patients were intubated because of insufficient respiration. Thirty patients out of 60 were extubated on the same day as surgery (Figure 4). Twelve were extubated the next day.

Of the 21 patients that were on a ventilator pre operatively, eight were extubated on the day of surgery. A further five were extubated the next day. Six of the pre operatively ventilator-treated patients needed ventilation for more than three days (Figure 4).

**Figure 5:**
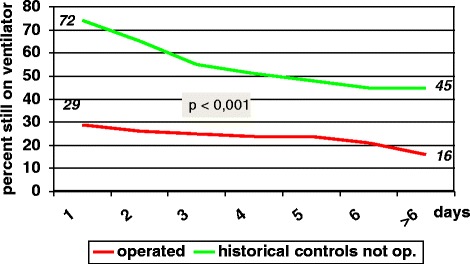
**Patients still on a ventilator during the first week: 153 patients with rib fractures who were not operated compared to 60 operated patients.**

Figure 5 shows the number of operated patients on the ventilator from day one post operatively and during the first week, together with our historical non operated controls. The difference between the groups is significant (p < = .0001).

There was a significant correlation between ISS and days spent in the mechanical ventilator (p<0.015). The difference in ISS between the controls and the operated patients was not significant, however.

### Infections

No clear pneumonia was found. We had one deep infection. This patient early developed a sinus that constantly came back in spite of continuous treatment with antibiotics. This infection progressed into osteomyelitis. Reoperation was performed after seven months. The patient was treated with antibiotics for another three months. The scar was tender at follow-up 12 months after the primary surgery, but no other signs or symptoms of infection were present. Defects in two ribs could be found on x-ray 15 months after the injury.

### Reoperations

Three patients were reoperated. Two plates were removed from the patient with the deep infection seven months after primary surgery. One patient was reoperated because of pulmonary leakage on post op day five, with a non anatomic resection of a lung segment. One patient was reoperated because of local tenderness at the site of a protruding plate. This was a highly trained athlete with sparse subcutaneous fat.

### Death

Two patients died during the acute period. One was a woman, 69 years old with fractures to the cervical spine, lumbar spines and pelvis. Her flail chest and ruptured diaphragm were operated on day 4 followed by the pelvic fracture. She died on day 24 because of multiple organ failure. The second case was a man, 71 years old with hypertonia, atrial flutter and COPD. He was operated on day 10 and died on day 30 because of pulmonary failure.

### Fracture complications

No early fracture dislocations or complications from the plates or splints could be found on standard x-rays.

### Costs

Median costs for the first 29 operated patients was 32 300 USD compared to 37 100 USD, median value, for 39 non-operated controls (difference 4800 USD).

## Discussion

Many different treatments were advocated before 1950^,^ when the use of the mechanical ventilator was gradually accepted [7]. There has been new interest in surgical treatment of traumatic rib fractures during the past decade. This might be due to a better understanding of the biomechanics of the ribs, better understanding of the fracture pattern of the chest, better implants of titanium and better surgical techniques. Multiple rib fractures and flail chest are present in 9% of high-energy trauma patients and (this is also true - ?) in our hospital (153/8680 patients over six years). This figure is the same in most centres dealing with this type of injury [1,8,20].

Mortality due to isolated rib fractures is low in our series and is dictated more by the patient’s general condition, age and other injuries [20]. The risk of mortality is increased in relation to the number of fractured ribs [20]. The condition of multiple rib fractures is also a marker of other severe injuries with a high mortality risk [1,21]. We had two deaths, one as an effect of MOF and one in an older man with pulmonary disease.

The risk for complications and death is increased in the elderly, and this might be the group that has the greatest benefit from surgical treatment, in whom the risk of an adverse outcome after four fractured ribs is significantly increased [4,22,23]. The proportion of older patients at risk is high in our study but is probably an indication of elderly persons’ problems in withstanding trauma. The proportion of osteoporosis is obvious for this group, Figure 1 [24].

Our figures for post operative time in a mechanical ventilator are low [2.7 days] compared to our historical controls as well as to other series. Tanaka [9] showed the longest time in a ventilator, 10.8 days, but also the highest ISS, 33. Althausen [13] had 4.14 days in a ventilator and an ISS of 25.1. We found 2.7 days in a ventilator and an ISS of 21.7. The time in a ventilator was only two days in Granetzny’s series [10], but he also had the lowest ISS, 16.8. Higher ISS indicates that there are serious injuries other than in the rib cage that are the indication for treatment in a ventilator. However, comparisons are also jeopardised by differences in age and gender. Figure 3 shows the difference, between the two series and gender compared to ISS. It is clear that the difference between the two series is due to more operations in the ISS group 16 to 20 irrespective of gender. The chest injury is dominant in this group. It is possible that these almost isolated chest injuries are the group that benefit most from surgery. The Abbreviated Injury Scale (AIS), which is the basis for calculating the ISS, has been revised three times since 1998, which makes comparisons of patients during different times uncertain [25]. One important difference is that head and intracranial bleeding has been less weighted in newer materials. The spread in ISS is also huge, 21.7 (SD +−10.8), for our study compared to 30.9 (SD+−13.3) for our historical controls. This is not a significant difference. The difference with our historical controls can also be questioned because of differing indications and policies. It is possible that we now send more patients back to local hospitals early. To avoid this bias, median values can be used instead of means. The median value for the operated group in our hospital is one (0–21) day as compared to the five days in the non operated group.

Our choice of the Matrix® system is based on the development of other fracture implants. The new implants with a locked angle between the plate and the screws have made it possible to fix even very osteoporotic fractures in other bones. The bowed rib is the ideal fractured bone for plating as the plate is positioned on the convex side of the bone, working as a true tension band. We therefore preferred the plates whenever possible. The modern plate fixating technique is demanding and requires proper training end education, as was pointed out by Mayberry in [26] in a comment to Althausen [13], who used the same implant. The splint has a less favourable mechanical position and does not offer as good stability as the plate. In our work, the splint has been used for single, less unstable fractures or when we could not use the plate for technical reasons. We could not find any early fracture dislocations or mechanical failures in our osteosynthesis.

The frequency of pulmonary infection is significantly lower after surgical treatment [9,13]. We could not find any clear clinical symptoms and radiological signs of pneumonia in our operated series. All patients had prophylactic antibiotics at the least until the day after the chest drains were removed. We also think that our thoracotomies, where we cleared out the pleura from haematoma and debris and resected lacerated lungs, added to our low rate of infection. Lung contusion is said to be a contra indication for rib fracture surgery [27]. In 11 out of 45 patients with CT-verified pulmonary contusion, we did some type of resection. This might have been a good way to exclude complications from the parenchyma. While we could not find any differences for the patients with lung contusion, we did not classify the extent of the contusion.

The costs as we have found them indicate that surgery could be favourable. The difference is not great. We wish to interpret this with caution and conclude that operating flail chest does not increase hospital costs. A prospective cost benefit analysis should be done that includes sicklisting time, benefits, loss in GDP and late disability.

## Conclusions

Open reduction and internal fixation is a safe method for treating the unstable thoracic cage with multiple rib fractures and flail chest.

The rate of early complications is low.

The need and time for treatment in a mechanical ventilator are significantly decreased.

Surgical treatment is probably cost effective and can be recommended.

Knowledge of thoracic surgery and modern fracture surgery is needed.

Intrapleural debridement might add to our good results.

This is a level 3 study from a consecutive cohort of historical controls there is still a lack of randomized studies comparing conservative and operative treatment. Studies on late disability after flail chest should also be undertaken.

### Consent

Written informed consent was obtained from the patient for the publication of this report and any accompanying images.
